# Development, Usability, and Quality Evaluation of a Mobile Application to Enhance the Functional Aspects of Social Relationships Among Cancer Patients: An Applied‐Developmental Mixed‐Methods Study

**DOI:** 10.1002/hsr2.71402

**Published:** 2025-10-22

**Authors:** Bahare Zarei, Masoud Bahrami, Hossein Beigi‐Harchegani, Ashraf Kazemi

**Affiliations:** ^1^ Department of Nursing, Faculty of Nursing and Midwifery Birjand University of Medical Sciences Birjand Iran; ^2^ Department of Adult Health Nursing, Nursing and Midwifery Care Research Center, Faculty of Nursing and Midwifery Isfahan University of Medical Sciences Isfahan Iran; ^3^ Department of Medical Library and Information Sciences, Health Information Technology Research Center, Faculty of Management and Medical Information Sciences Isfahan University of Medical Sciences Isfahan Iran; ^4^ Department of Midwifery and Reproductive Health, Nursing and Midwifery Care Research Center, Faculty of Nursing and Midwifery Isfahan University of Medical Sciences Isfahan Iran

**Keywords:** Cancer, Mobile app rating scale, Mobile application, Social relationships, System usability scale

## Abstract

**Background and Aims:**

Beyond the physical burden of illness, cancer patients often face significant disruptions in their functional social relationships, profoundly impacting their overall well‐being. This study aimed to develop, assess usability, and evaluate the quality of a mobile application designed to address these perceived relational challenges.

**Methods:**

This study was conducted in four sequential phases. First, a systematic review and qualitative meta‐synthesis were carried out to identify the functional social relationship needs of cancer patients. These needs were subsequently prioritized and validated through expert consensus via the Delphi technique and panel discussions. On the basis of the validated needs, general and specific objectives were formulated, and educational content was systematically developed through an extensive literature review, and then refined and validated by the research team. An m‐Health application was designed and developed by integrating the validated content into a structured digital platform. Finally, usability and quality were assessed via the System Usability Scale and the Mobile App Rating Scale. Data analysis was performed in SPSS with both descriptive and inferential statistical methods.

**Results:**

The application was developed with six modules. Usability testing from the patients' perspective yielded a mean score of 88.62 (95% CI: 81.97–95.27), reflecting high satisfaction, strong perceived usefulness, and a high likelihood of recommendation to other cancer patients. Moreover, the app received an overall mean score of 4.15 (SD = 0.21), indicating a good‐quality design with minimal variability among reviewers.

**Conclusion:**

The developed m‐Health application demonstrated high usability and effectiveness in enhancing perceived aspects of social relationships among cancer patients. Positive evaluations from both patients and experts highlight its potential as a supportive tool for addressing relational challenges in oncology care.

## 1 Introduction

Cancer remains the second leading cause of death worldwide [1], with its global incidence steadily increasing in recent years [2]. According to projections from the World Health Organization (WHO), the number of cancer cases is expected to increase by nearly 50% by 2040 [3]. This alarming trend underscores the urgent need for a comprehensive, multifaceted approach to care that addresses the wide range of challenges faced by cancer patients.

Over the past three decades, increasing recognition has been given to the multifactorial determinants of health, including psychological, societal, sociocultural, political, economic, and environmental influences. This paradigm shift has led to a transition from a predominantly disease‐centered healthcare model to a more holistic, patient‐centered framework [4]. This approach emphasizes the integration of biomedical treatment with emotional, social, and environmental considerations in both clinical practice and health policy [5].

Within the context of cancer, this comprehensive perspective is particularly critical, given the profound burden of the disease and its far‐reaching impact on patients' lives. While considerable research has focused on the physical challenges of cancer, leading to major advances in medical interventions, the psychosocial dimensions, particularly those related to social relationships, have received comparatively limited attention. A review of global literature highlights this gap, revealing an insufficient emphasis on the role of social relationships in cancer care models. This oversight underscores the need to integrate social relationship considerations alongside other key dimensions of patient well‐being.

Social relationships refer to meaningful connections that individuals form through repeated interactions with family members, friends, neighbors, and acquaintances [6]. Scholarly research on social relationships has focused primarily on two dimensions: structural and functional. The structural dimension pertains to the quantitative and observable characteristics of an individual's social network, such as the number of close relationships, the nature of these connections, and the frequency of interactions. In contrast, the functional dimension emphasizes the qualitative nature of social interactions, highlighting their emotional significance and impact on an individual's well‐being [6, 7, 8]. This functional perspective extends beyond the mere presence of relationships, focusing on whether these connections provide support and enrichment or, conversely, contribute to stress and emotional distress. It also encompasses both positive and negative social experiences, which collectively shape an individual's overall social well‐being [6, 7, 8, 9].

As a fundamental determinant of health, social relationships influence both physical and mental well‐being through multiple mechanisms. First, they mitigate psychological stress and promote mental health by fostering a sense of belonging, providing emotional support, and enhancing coping strategies. Second, they regulate physiological systems, including the hypothalamic‒pituitary‒adrenal axis, cardiovascular function, and immune responses, thereby modulating biological reactions to stress and reducing the risk of chronic diseases. Third, social networks shape health behaviors by reinforcing positive habits, such as physical activity, while discouraging harmful behaviors, such as smoking [7, 10]. These pathways demonstrate how both the quality and the structure of social relationships affect health outcomes, including longevity and susceptibility to disease. However, not all social interactions are beneficial; certain relationships may contribute to social distress, rejection, or the reinforcement of unhealthy behaviors [11].

Cancer profoundly affects patients' social lives, with their experiences shaped by an intricate combination of personal circumstances, social contexts, and systemic factors. Misconceptions and stigma associated with cancer often result in social isolation, whereas insufficient support networks may fail to address patients' changing emotional and practical needs [12]. Many individuals report feelings of loneliness and social exclusion, which can profoundly affect their mental health and overall quality of life [13, 14]. Concern over negative social reactions may further discourage open communication about the illness, straining familial and social connections and, in some cases, prompting withdrawal from social interactions [15]. Interpersonal relationships, particularly with spouses, family members, and close friends, can present substantial challenges, illustrating the disruptive influence of cancer on social dynamics [16]. Complications in marital and sexual relationships may further compromise the stability and emotional well‐being of intimate partnerships [15]. Considering these difficulties and given evidence that disruptions in social relationships are strongly associated with adverse mental health outcomes and diminished quality of life in cancer patients [17, 18, 19], the implementation of targeted interventions to support social functioning is increasingly essential.

In recent years, digital health technologies have emerged as a transformative means of enhancing access to cancer care for both patients and healthcare providers [20]. Although digital health interventions originated in the 1920s, their evolution has accelerated markedly in recent decades, particularly through mobile applications [21]. These advances have produced accessible, cost‐effective tools that enable patients to manage their health more efficiently and, importantly, to navigate areas of vulnerability with increased confidence [22, 23, 24, 25].

A review of prior research indicates that most digital health interventions for cancer patients have predominantly targeted physical and psychological well‐being. However, despite the crucial role of social relationships in overall patient well‐being, this dimension has received comparatively limited attention. Given that social support and meaningful interpersonal interactions are essential for adapting to illness, there is a pressing need for interventions specifically addressing social functioning. However, designed digital health technologies offer innovative opportunities to foster and strengthen the social connections of cancer patients, thereby improving their quality of life.

Moreover, an analysis of various databases revealed that social care interventions, whether technology‐assisted or not, have generally been categorized under the broader umbrella of psychosocial interventions, with the primary aim of enhancing patients' quality of life and social well‐being [26, 27, 28]. Many of these interventions emphasize social support as a central component [29, 30]. Given the substantial number of cancer patients and the challenges they faced, especially concerning social relationships, along with the urgent need for innovative, technology‐based interventions, this study was conducted to design, assess usability, and evaluate the quality of a mobile application intended to enhance the functional aspects of social relationships among individuals diagnosed with cancer.

## 2 Methods

### 2.1 Study Phases

This applied‐developmental mixed‐methods study was conducted in four sequential phases, outlined as follows.

#### 2.1.1 The First Phase‐ Needs Identification and Prioritization

A systematic review and qualitative meta‐synthesis were performed to identify the functional social relationship needs of cancer patients. To ensure a comprehensive evaluation of studies on social relationships and mobile applications in the context of cancer, a systematic search strategy was employed. Initially, relevant keywords were identified and refined in alignment with the study objectives. A thorough search was conducted across multiple international databases, including PubMed, Web of Science, Scopus, and ScienceDirect, as well as national databases such as SID, Noormags, and Magiran. The search period spanned from 1900 to March 2024, encompassing both historical and contemporary research. To enhance search precision, MeSH‐compliant keywords were utilized, covering terms related to cancer, social relationships, interpersonal relationships, social support, loneliness, social alienation, and rejection. Boolean operators (“OR” and “AND”) were strategically applied to optimize search term combinations across categories. Additionally, pilot searches were conducted to refine keyword selection, and a reference guide on social relationships from the National Institutes of Health (NIH) was consulted [9]. Furthermore, mobile applications available in both international and domestic app marketplaces were systematically reviewed to assess their relevance to the social relationship needs of cancer patients. The identified needs were then prioritized and validated through the Delphi technique and expert panel consensus. Panel members were selected via purposive and snowball sampling to ensure the inclusion of a diverse and knowledgeable group. The panel comprised ten experts, including researchers, a psycho‐oncologist, two psychiatric nurses, a psychologist, and other professionals specializing in cancer patient care.

#### 2.1.2 The Second Phase‐ Content Preparation

Based on the validated needs, both general and specific objectives were established, and the educational content was systematically developed through a rigorous and comprehensive literature review. To ensure scientific accuracy, clinical relevance, and practical applicability, an extensive search was conducted across specialized cancer‐related websites, authoritative textbooks, and peer‐reviewed articles within both national and international academic databases. The content development process adhered to evidence‐based principles, integrating the latest research findings and best practices in psycho‐oncology, patient education, and supportive care for cancer patients. Information was synthesized from reputable oncology and psycho‐oncology associations, leading healthcare institutions, and professional guidelines, ensuring that the material effectively addressed the social relationship challenges faced by cancer patients. The compiled content underwent iterative refinement through research team discussions, where it was rigorously reviewed and validated. This validation process ensured scientific rigor, cultural relevance, and real‐world applicability, enhancing its effectiveness in improving the social well‐being of cancer patients.

#### 2.1.3 The Third Phase‐ App Development

The mobile app was developed based on validated requirements. The app features a single user interface (UI) designed specifically for cancer patients, ensuring ease of use and accessibility. The application was developed using Java within the Android Studio environment, following a modular programming approach to enhance code maintainability, reusability, and flexibility [31]. For data management, the app does not utilize a central database; instead, it employs Shared Preferences to store user information in a key‐value format within an XML file, ensuring lightweight storage and efficient access. However, in the entertainment module, SQLite was integrated to handle structured data. The UI design was carefully crafted to optimize patient engagement, considering usability principles and color psychology to create a visually appealing and emotionally supportive environment [32]. The app is named *Peyvandi No*, which means new connections and links in Persian.

#### 2.1.4 The Fourth Phase‐App Evaluation

To assess the usability and quality of the *Peyvandi No* app, two complementary evaluation methods were employed: patient (end‐user) and expert evaluation.

▪ **Patient Participants:** For the patient evaluation, usability assessment was conducted based on direct user feedback. A member of the research team (the first author) was stationed at cancer treatment centers in Isfahan and the Iranian Cancer Control Center (MACSA) to engage with patients, explain the study objectives, and outline participation requirements. Eligible patients were invited to participate in the usability evaluation, with selection based on convenience sampling and predefined inclusion criteria. To qualify, participants had to be at least 18 years old, possess an Android‐based smartphone, hold Iranian nationality, and have sufficient physical, mental, and social capability to use the app effectively. Patients who did not complete the usability assessment questionnaire were excluded from the study. Upon expressing their willingness to participate, patients were provided with a detailed explanation of the study's purpose and potential benefits. The *Peyvandi No* app was then installed on their smartphones, and they received in‐person training on how to navigate its features. Once the successful installation was confirmed, patients were instructed to use the app regularly over a 5‐week period. To enhance engagement and encourage consistent usage, daily motivational reminder messages were sent to participants, and biweekly follow‐up calls were made to monitor their experiences and address any issues. At the end of the 5‐week period, participants were asked to complete an online survey, which included a demographic questionnaire and the System Usability Scale (SUS). SUS, introduced by Brooke (1996), is a standardized survey scale for evaluating the usability of a product or service. Recognized as a reliable, quick, and easy‐to‐use method, SUS provides a single usability score that is easily interpretable across a wide range of users. This tool is also flexible enough to assess the usability of various products and services. Research has demonstrated that SUS is a dependable instrument for measuring usability [33]. The SUS questionnaire consists of ten five‐point Likert‐scale statements, each addressing different aspects of usability. These statements are rated on a scale from 1 (strongly disagree) to 5 (strongly agree). Five of the items are positively worded (odd‐numbered items), while the other five are negatively worded (even‐numbered items). The overall SUS score is calculated by subtracting 1 from the score of each odd‐numbered item and subtracting the score of each even‐numbered item from 5. The adjusted scores are then summed and multiplied by 2.5, yielding a final SUS score ranging from 0 (very poor usability) to 100 (excellent usability). A SUS score of 80.3 or higher indicates that the mobile app is well‐received, users like it, and they would recommend it to others. A SUS score of 68 or higher suggests that the app is considered good but may require some improvements. However, a SUS score below 68 signifies poor usability, indicating that substantial enhancements are needed [34].
▪ **Expert participants:** In addition to patient feedback, an expert evaluation was conducted using the Mobile App Rating Scale (MARS), a widely recognized tool for assessing the quality of m‐Health applications. In this study, three experts specializing Medical Informatics and Software Engineering were selected, each with a minimum of 3 years of experience in e‐health, healthcare system evaluation, and mobile app development. The MARS is a standardized tool designed to evaluate the quality of mobile applications from an expert perspective. It consists of 29 items categorized into four key objective subscales: engagement, functionality, esthetics, and information. The engagement subscale (items 1–5) assesses user interest, interactivity, customization, and overall appeal. The functionality subscale (items 6–9) evaluates usability, smooth operation, technical performance, and adaptability. The esthetics subscale (items 10–12) examines visual design, graphical quality, and overall esthetic appeal. The information subscale (items 13–19) measures the accuracy, clarity, timeliness, credibility, and relevance of the provided content. In addition to these objective subscales, MARS includes a subjective quality subscale (items 20–23) that captures users' perceived overall quality of the app. It also features a perceived impact section, consisting of six additional items that assess the app's influence on users' knowledge, awareness, and motivation to engage in target behaviors. All MARS items are rated on a five‐point Likert scale. The objective subscales are scored from 1 (poor) to 5 (excellent), while perceived impact items are rated from 1 (strongly disagree) to 5 (strongly agree). A “Not applicable” option is available for items that may not be relevant to all apps. Each subscale score is calculated by averaging the ratings of its respective items, and the overall app quality score is derived from the average of the four objective subscale scores. MARS is widely recognized as a valid and reliable instrument for evaluating digital health applications. It provides valuable insights for researchers, developers, and healthcare professionals, helping to enhance app quality, improve user experience, and ensure effectiveness in health interventions [35].

### 2.2 Data Analysis

The data were analyzed via SPSS software version 23.0 (IBM Corp., Armonk, NY, USA). Descriptive statistics included means, standard deviations (SDs), frequencies, and percentages. The normality of continuous variables was assessed with the Shapiro–Wilk test. Since the distributions of the main variables deviated from normality, nonparametric tests were applied. Specifically, the Mann–Whitney U test was used for binary variables, and the Kruskal–Wallis test was applied for variables with more than two groups. All tests were two‐sided, with a predefined significance level of *p* < 0.05. In addition to P values, effect sizes and 95% confidence intervals (CIs) were reported, where applicable, to indicate the magnitude and precision of the observed effects.

### 2.3 Ethical Considerations

The Ethics Committee of IUMS (Isfahan University of Medical Sciences), Iran, approved the study protocol (Approval Code: IR. MUI. NUREMA. REC.1402.081). Before the app installation, all patients were informed about the study's objectives, procedures, potential outcomes, and associated risks. Written informed consent was obtained from all participants before their inclusion in the study.

## 3 Results

### 3.1 First and Second Phases

The *Peyvandi No* structure is based on findings from a qualitative meta‐synthesis and a systematic quantitative review. After prioritization and validation by an expert panel, the most critical social needs were identified and integrated into the application to offer a structured, evidence‐based response to the social challenges patients face. The core dimensions, identified needs, representative content component, and their respective delivery formats are outlined in Table 1.

**Table 1:** Content structure of the mobile application for enhancing functional aspects of social relationships.

Core dimension	Identified need	Content components	Delivery format
Marital and intimate struggles during illness	Fostering emotional and sexual intimacy	– Understanding the psychological and relational impact of illness on marital intimacy – Enhancing emotional and sexual connection despite health‐related challenges – Developing communication strategies for intimacy and mutual understanding	– Educational texts – Videos – Shared experiences – Practical exercises – Feedbacks of self‐evaluation and interactive engagement
Discrepancies and perceived inadequacies in social support	Developing effective strategies for conflict resolution	– Identifying common sources of interpersonal conflict – Conflict resolution techniques tailored to illness‐related stressors – Effective negotiation and boundary‐setting within personal relationships	– Educational texts – Practical exercises – Feedbacks of self‐evaluation and interactive engagement
Challenges of social isolation and reintegration	Cultivating acceptance of life circumstances while strengthening resilience	– Psychological adaptation to changes in life and social roles – Strategies for maintaining social identity and self‐worth – Coping mechanisms for managing loneliness and isolation	– Educational texts – Videos – Shared experiences – Practical exercises – Feedbacks of self‐evaluation and interactive engagement
	Reorganizing social relationships to align with current circumstances	– Reassessing personal goals and expectations for social participation – Identifying and overcoming challenges in social interactions – Building and maintaining supportive social networks – Enhancing readiness for social reintegration, both physically and emotionally – Strengthening communication and empathy skills for meaningful relationships	– Educational texts – Videos – Shared experiences – Practical exercises – Feedbacks of self‐evaluation and interactive engagement
	Adjusting to changes in physical appearance due to cancer	– Psychological strategies for body image resilience – Coping with altered self‐perception and societal reactions – Enhancing self‐confidence through self‐care and adaptive strategies	– Educational texts – Animation – Shared experiences – Practical exercises – Feedbacks of self‐evaluation and interactive engagement
Social support	Sustaining continuous understanding and social support from family and friends	– Identifying emotional and practical support needs – Communicating limitations and needs effectively to loved ones – Strengthening and maintaining close relationships with family and friends – Enhancing the ability to seek and accept support	– Educational texts – Shared experiences – Practical exercises – Feedbacks of self‐evaluation and interactive engagement
Understanding stigma and rejection	Reducing the sense of isolation from the healthy community	– Strengthening self‐worth and social identity beyond illness and physical changes – Raising awareness of societal misconceptions about cancer – Developing skills to navigate judgment and intrusive questions – Managing energy levels effectively for social engagement – Setting realistic expectations regarding personal abilities – Encouraging participation in volunteer work, support groups, or community activities	– Educational texts – Videos – Shared experiences – Practical exercises – Feedbacks of self‐evaluation and interactive engagement
Challenges in seeking support	Alleviating feelings of burden and dependency	– Recognizing and addressing the root causes of feeling like a burden – Enhancing open communication to express emotions and needs transparently to family – Restoring a sense of independence and purpose by maintaining personal responsibilities and meaningful engagement – Challenging negative self‐perceptions and fostering a balanced sense of contribution in relationships	– Educational texts – Feedbacks of self‐evaluation and interactive engagement
Interpersonal tensions arising from excessive pity and misguided sympathy	Experiencing empathy rather than misguided sympathy or pity	– Managing conversations and emotions in illness‐related discussions – Effectively expressing needs and feelings to family and close contacts – Gaining realistic insight into others’ emotions and intentions – Reframing sympathy and pity for a more constructive social response	– Educational texts – Feedbacks of self‐Evaluation and interactive engagement
Challenges in disclosing information	Managing the disclosure of personal information	– Classifying illness‐related information based on relevance and necessity – Assessing appropriate levels of disclosure according to the audience – Developing skills for controlled and healthy disclosure in conversations	– Educational texts – Feedbacks of self‐evaluation and interactive engagement

### 3.2 Third Phase

**Figure 1:**
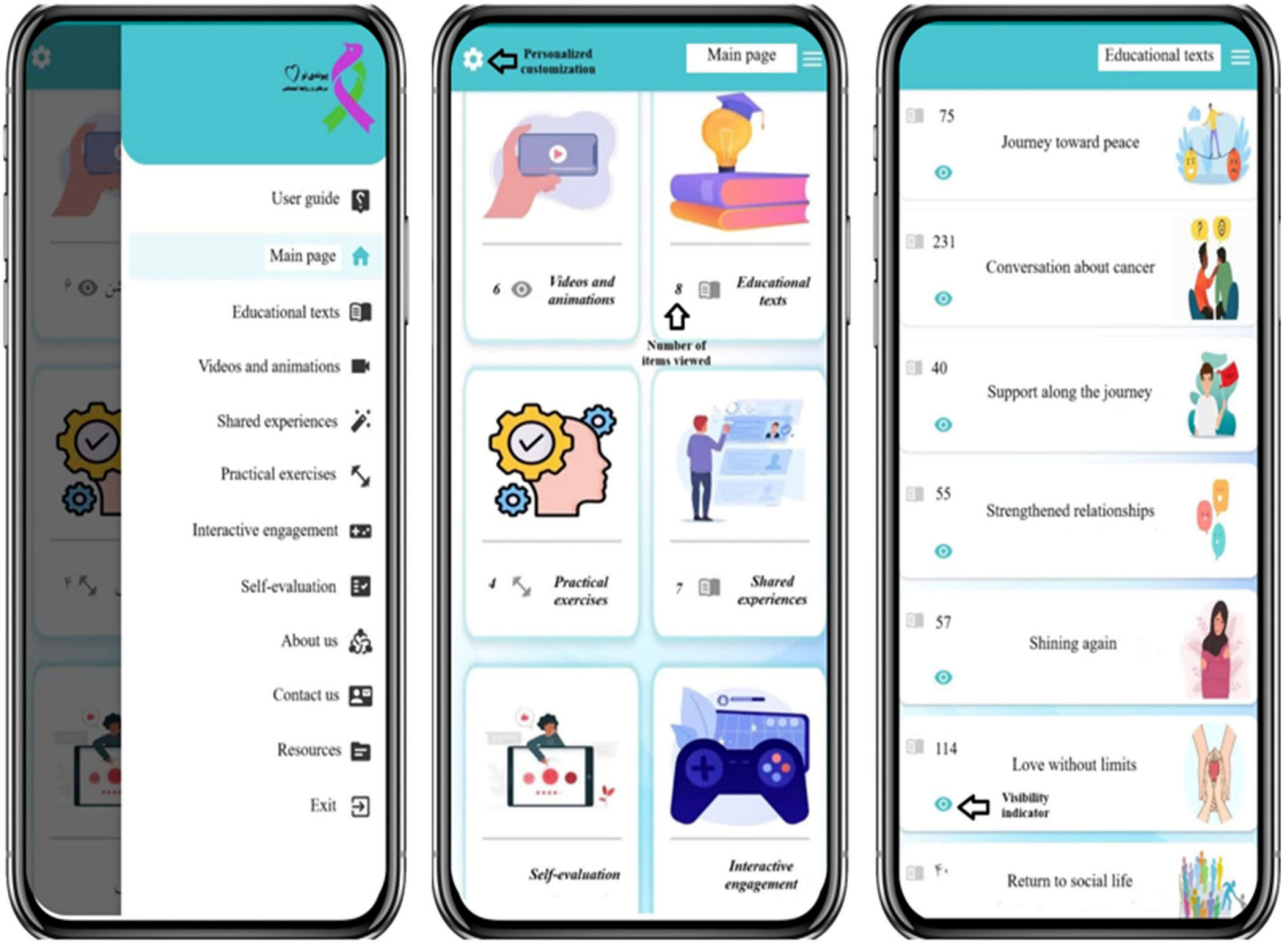
The main interface of the *Payvandi No* app, displaying core modules and navigation options.

The *Peyvandi No* application comprises six core modules: educational texts, videos and animations, shared experiences, practical exercises, self‐evaluation, and interactive engagement. Upon installing the software, users are immediately directed to the main interface, which presents these modules without requiring registration or login, ensuring seamless access to content. A right‐side panel facilitates navigation to essential sections, including the User Guide, Modules, About Us, Contact Information, Resources, and Exit Button, providing an intuitive user experience. Each module or educational section begins with a brief instructional guide, ensuring clarity before engagement. The entire application functions offline, allowing users to access content without an internet connection. In sections featuring textual content, a visibility indicator (eye icon) changes color once the text has been read, providing a visual progress tracker. Furthermore, the system logs and displays the number of completed readings and viewed videos, enabling users to monitor their progress effectively. Additionally, a setting icon at the top of the main screen allows for personalized customization, including theme color selection, font adjustments, and background music control, enabling users to tailor their experience on the basis of individual preferences (Figure 1).

**Figure 2:**
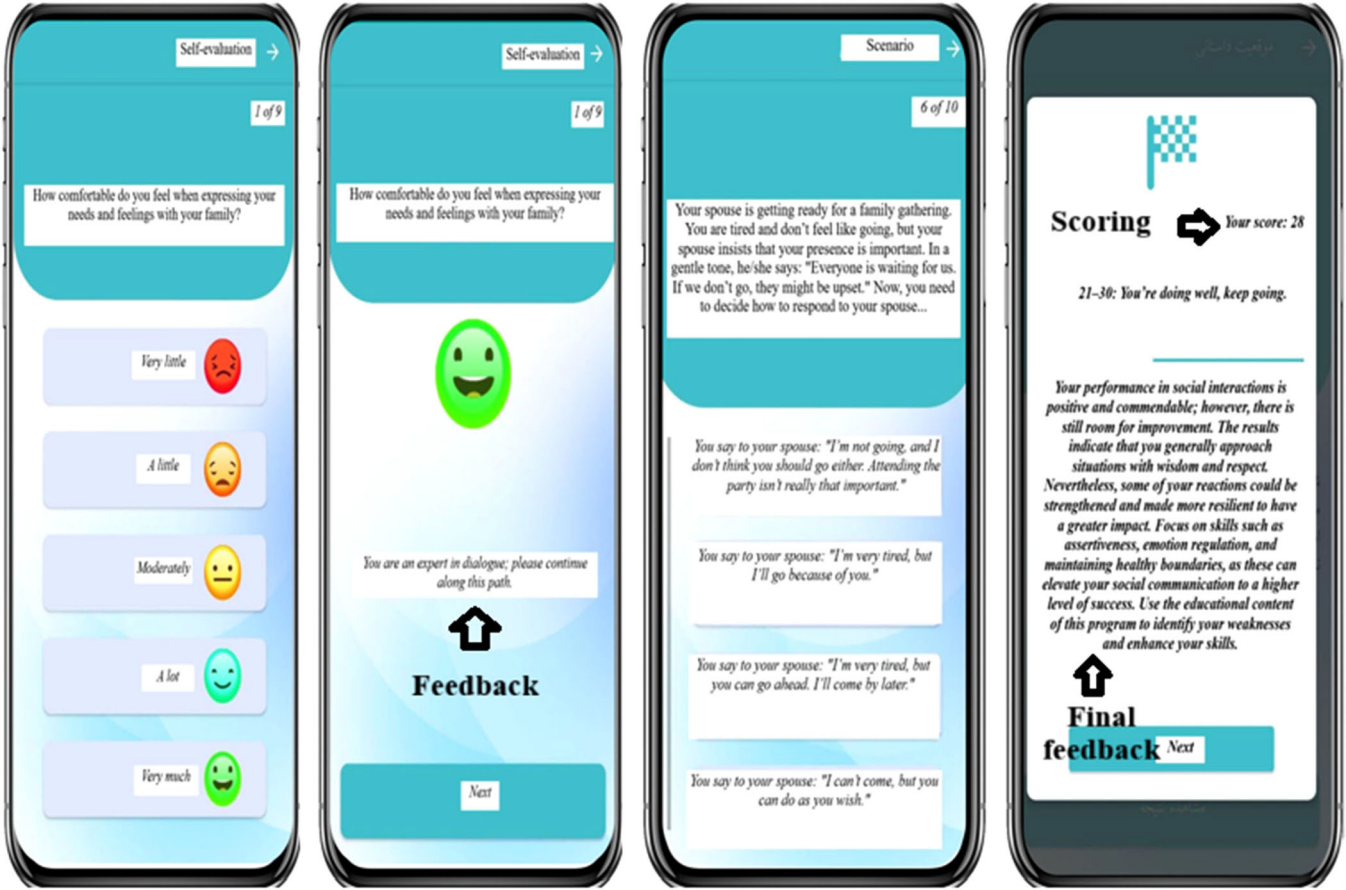
Self‐evaluation and interactive engagement modules of *Payvandi No* app, featuring scenarios, questions, feedback, and scoring.

The self‐evaluation and interactive engagement modules incorporate interactive scenarios and quizzes designed to provide real‐time feedback and cumulative scoring, fostering an engaging and dynamic learning experience (Figure 2).

### 3.3 Fourth Phase

Twenty cancer patients participated in the usability evaluation, with all (100%) providing consent for the end‐user assessment. The participants had a mean age of 36.45 years (SD = 9.80) and an average time since diagnosis of 21.80 months (SD = 13.01). Additional demographic characteristics are summarized in Table 2.

**Table 2:** Demographic characteristics of cancer patients (end‐users).

		Frequency (*n*)	Percentage (%)
Gender			
	Female	8	40
	Male	12	60
Marital status			
	Single	7	35
	married	13	65
Job			
	Employed	8	40
	Homemaker	4	20
	Retired	1	5
	unemployed	7	35
Education			
	Less than high school	4	20
	High school diploma	8	40
	Higher education	8	40
Place of residence			
	Urban	15	75
	Rural	5	25
Financial status			
	Below average	5	25
	Average	14	70
	Above average	1	5
Treatment type			
	Chemotherapy	1	5
	Surgery	0	0
	Radiotherapy	1	5
	Combined	18	90
Currently undergoing treatment			
	Yes	17	85
	No	3	15
Age (years)			
	18–32	7	35
	33–46	10	50
	> 47	3	15
Time since diagnosis (months)			
	6‐18	11	55
	19‐31	5	25
	> 32	4	20

The results of patients' evaluations, presented in Table 3, indicate an SUS score of 88.62 (out of 100), reflecting a high level of user satisfaction with the application's usability and a strong likelihood of it being recommended to other cancer patients. The participants reported ease of use, quick learnability, and high confidence in using the application, with minimal complexity or need for technical support. Furthermore, the mean SUS score for this application (SD = 14.19, 95% CI: 81.97–95.27) was significantly higher than the normative mean of SUS = 68 (*p* < 0.001). Here, the reported p value tests the null hypothesis that the mean SUS score of our participants is equal to the normative SUS score of 68, confirming the application's superior usability and effectiveness compared with standard benchmarks.

**Table 3:** Evaluation of SUS (*N* = 20).

No.	Statements	Mean scores	SD
1	Like to use the application frequently	4.30	0.97
2	The application is unnecessarily complex	1.40	0.75
3	The application is easy to use	4.40	0.82
4	Need technical support to use the application	1.55	0.60
5	Various functions in this application are well integrated	4.35	0.87
6	Too much inconsistency in the application	1.35	0.74
7	Learn to use the application very quickly	4.65	0.81
8	The application is very cumbersome to use	1.15	0.36
9	Very confident using the application	4.70	0.57
10	Need to learn a lot before using the application	1.50	0.82
Adjusted Even‐Item Score (25 − Sum of even items)		17.40	3.28
Adjusted Odd‐Item Score (Sum of odd items − 5)		18.05	2.68
Total SUS Score (Sum of converted items)		35.45	5.67
Final SUS Score (Total SUS Score × 2.5)		88.62	14.19

Normality tests via the Shapiro‒Wilk and Kolmogorov‒Smirnov tests revealed a non‐normal SUS score distribution (*p* < 0.05). Consequently, non‐parametric methods were applied to assess variations across demographic variables.

The Mann‒Whitney U test was conducted for binary demographic variables, including marital status, place of residence, and current treatment status (Table 4), whereas the Kruskal‒Wallis test was applied to variables with more than two groups, such as age, employment status, education level, financial status, treatment type, and time since diagnosis (Table 5). Effect sizes were calculated for all comparisons (*r* for the Mann‒Whitney U test; η² for the Kruskal‒Wallis test) to provide a measure of the magnitude of differences independent of sample size.

**Table 4:** Mann–Whitney U test comparing SUS scores between two independent demographic groups.

Variable	*Z*	Sig. (2‐tailed)	*r*
Marital status	−0.16	0.87	0.035
Place of residence	−1.05	0.30	0.234
Currently undergoing treatment	−1.22	0.25	0.272

**Table 5:** Kruskal–Wallis test comparing SUS scores across multiple demographic groups.

Variable	Chi‐square	*df*	Sig. (2‐tailed)	*η²*
Age	0.11	2	0.94	0.111
Job	2.73	3	0.43	0.017
Education	1.92	2	0.38	0.005
Financial status	0.64	2	0.72	0.080
Treatment type	0.66	2	0.71	0.079
Time since diagnosis	0.60	2	0.74	0.082

As shown in Table 4, no statistically significant differences were observed for any binary demographic variable (all *p* > 0.05). The effect sizes ranged from very small (*r* = 0.035 for marital status) to small (*r* = 0.272 for current treatment status), indicating minimal practical differences in SUS scores across these groups.

Similarly, Table 5 shows that multigroup variables also exhibited no statistically significant differences in SUS scores (all *p* > 0.05). The corresponding effect size values ranged from 0.005 (education) to 0.111 (age), reflecting negligible to small effect sizes. These results suggest that participants' perceptions of system usability were consistent regardless of age, employment status, education level, financial status, treatment type, or time since diagnosis. Overall, the findings indicate that demographic characteristics had minimal impact on SUS scores and that differences in perceived usability across demographic subgroups were practically insignificant.

Table 6 presents the evaluation results of the Mobile App Rating Scale (MARS) based on assessments from three evaluators (*N* = 3). Among the different subscales, the highest score was observed in the app‐specific category (*M* = 5.00, SD = 0.00), indicating unanimous agreement among evaluators that the app effectively fulfills its intended purpose. In contrast, the lowest score was assigned to the subjective quality subscale (*M* = 3.08, SD = 0.14), suggesting that while the app performs well in objective measures, users may not perceive it as highly engaging or satisfying overall.

**Table 6:** Evaluation of MARS (*N* = 3).

	Mean score	SD
A: Engagement	3.33	0.28
B: Functionality	4.50	0.38
C: Esthetics	4.44	0.38
D: Information	4.33	0.28
E: Subjective quality	3.08	0.14
F: App‐specific	5	0
App quality (total score)	4.15	0.21

Among the objective subscales, functionality (*M* = 4.50, SD = 0.38) and esthetics (*M* = 4.44, SD = 0.38) received high ratings, demonstrating the app's strong usability, intuitive navigation, and visual appeal. Similarly, information quality (*M* = 4.33, SD = 0.28) was rated positively, indicating that the content was clear, reliable, and well structured. However, engagement (*M* = 3.33, SD = 0.28) received a relatively lower score, implying that the app may lack interactive elements or features that encourage sustained user involvement. The overall app quality score (*M* = 4.15, SD = 0.21) suggests that, despite minor limitations, the app is generally well designed and functional.

## 4 Discussion

In this study, we developed and evaluated *Peyvandi No*., a mobile application designed to enhance the functional aspects of social relationships among cancer patients. *Peyvandi No* integrates strategies to foster emotional and sexual intimacy, implement effective conflict resolution mechanisms, promote illness acceptance, and strengthen resilience, offering a comprehensive framework for improving patients' interpersonal dynamics. Additionally, the app educates users on reorganizing social relationships, adapting to physical changes, and maintaining strong social support from family and friends. By reducing feelings of isolation and dependency, fostering authentic empathy, and providing strategies for managing health‐related disclosures, *Peyvandi No* stands out as a digital psychosocial intervention for cancer patients.

A systematic search was conducted to identify applications comparable to *Peyvandi No*. The findings revealed that no existing app specifically educates cancer patients on functional social relationships in such a comprehensive manner. However, several apps incorporate elements related to functional social relationships. For instance, the MGCS, designed for patients with gynecologic cancers, primarily emphasizes supportive care while also offering education on sexual problems and providing emotional support [36]. Likewise, iaya, a more generalized platform for various cancer types, facilitates social support and experience‐sharing among patients [37].

Further comparisons revealed that applications such as BCS, Becca, and Bezzy BC, which were specifically developed for breast cancer patients, prioritize networking with healthcare professionals and peer support [38, 39, 40]. LivingWith: Cancer Support serves as a social networking and communication management tool, whereas CancerAid and CanSurround focus primarily on psychosocial support and coping strategies. However, these apps emphasize patient education and long‐term follow‐up care [41, 42, 43]. Additionally, My Cancer Coach provides education on communication skills, spousal relationships, sexual health, body image, loneliness reduction, and mental well‐being [44]. A self‐management mobile app for women with breast cancer also addresses key aspects such as social avoidance, negative emotions, psychological distress, and body image concerns, aligning with the themes explored in the study [45].

The *Peyvandi No* app comprises six core modules: educational texts, videos and animations, shared experiences, practical exercises, self‐evaluation, and interactive engagement. This comprehensive design integrates multimedia elements, including text, video, audio, graphics, animation, and interactivity, creating a dynamic and engaging learning experience. The significant role of multimedia in teaching and learning is widely acknowledged. Multimedia‐enhanced courseware is recognized as an effective tool for engaging learners, particularly in subjects involving theoretical concepts. Multimedia refers to the integration of diverse digital media types, such as text, images, sound, and video, into an interactive, multisensory application or presentation designed to communicate information effectively [46]. Multimedia learning systems that incorporate animation and narration serve as powerful instruments for enhancing learners' comprehension [47].

To enhance user engagement, the app's design incorporates principles of color psychology tailored for interactive applications intended for patients. Personalization features, including customizable color themes, fonts, and background music, have also been integrated. These elements facilitate user‒software interaction, improve visual appeal, and ensure the use of appropriate color schemes [48]. In the self‐evaluation module, facial emojis are used for response selection. Research indicates that using facial emoji scales for feedback can significantly improve user engagement [49, 50]. Furthermore, the app's ability to accommodate the specific needs of cancer patients with certain limitations plays a crucial role in its usability [51]. An audio playback feature for textual content further supports accessibility and ease of use.

The usability assessment revealed an SUS score of 88.62 out of 100, indicating a high level of user satisfaction and a strong likelihood that the app would be recommended to other cancer patients. These findings are consistent with the usability scores of other m‐Health applications designed for cancer care, highlighting the app's user‐centered design and practical effectiveness. For comparison, the iaya app achieved an SUS score of 73.7 [37], the CaRA app scored 83.8 [52], and a self‐management mobile app for breast cancer patients received an SUS score of 83 [45], further supporting the strong usability of *Peyvandi No*. According to app evaluation studies, m‐Health applications exert the greatest impact when developed in accordance with standard usability and functionality criteria, which in turn enhances user adoption and engagement [53, 54].

No significant differences were observed in SUS scores across demographic variables such as age, gender, education, and financial status, suggesting that the app's user‐centered design effectively accommodates a diverse user base. This finding aligns with the principles of usability engineering, which emphasize the development of intuitive and accessible digital environments for users with varying needs [55]. One possible explanation is the app's intuitive interface and comprehensive in‐app tutorials, which help bridge gaps for users with different levels of digital literacy. Research indicates that psychological factors, such as attitudes toward technology, prior digital experience, and individual motivation, have a greater influence on user acceptance than demographic characteristics [56, 57]. Furthermore, the narrowing digital divide in recent years has contributed to more consistent engagement with health‐related technologies across diverse demographic groups [58]. Collectively, these findings underscore the importance of designing digital health interventions that are universally accessible, suggesting that *Peyvandi No*. has, to some extent, implemented inclusive design principles to meet the needs of a broad spectrum of users.

The overall MARS score of 4.15 suggests that the application maintains a good standard of quality, effectively balancing usability, esthetics, and information reliability. Among the four key objective subscales, functionality received the highest rating, indicating that the app performs its intended tasks efficiently and provides a seamless user experience. This strong functionality reflects well‐structured navigation, intuitive interface design, and reliable technical performance. However, further enhancements, such as the incorporation of smart features like advanced search options, personalized settings, or AI‐driven recommendations, could refine user experience and efficiency. The esthetics subscale also received a high score, highlighting the app's visually appealing interface and well‐organized design elements. While this suggests that the graphical presentation aligns with user expectations, implementing more dynamic visual components, such as adaptive themes, user‐customizable layouts, or animated transitions, may further enhance user engagement. In contrast, the engagement dimension received the lowest score, indicating that users may not feel sufficiently motivated to use the app consistently. This finding underscores the need to enhance interactive elements and emotional engagement. Strategies such as gamification, personalized user journeys, and dynamic feedback mechanisms could significantly improve user retention. For instance, incorporating progress tracking, reward‐based incentives, or community‐driven interactions (e.g., discussion forums or peer support networks) may encourage sustained app usage. The information quality rating suggests that the app provides credible and useful content. Since the application already provides linking to authoritative external resources, this feature enhances its credibility and reliability. However, ensuring comprehensive coverage of key topics and diversifying content formats could enhance its educational value. Integrating multimedia elements, such as instructional videos, interactive infographics, and voice‐guided explanations, may cater to different learning preferences and improve accessibility. Beyond the four primary subscales, the subjective quality score reveals a potential gap between technical excellence and user satisfaction. Despite strong functional and esthetic attributes, users may not perceive the app as fully engaging or fulfilling their expectations. Addressing this discrepancy requires a user‐centered design approach that incorporates personalized experiences, adaptive content recommendations, and intuitive onboarding processes to enhance perceived value. The app‐specific score reflects unanimous agreement among evaluators that the application successfully fulfills its intended purpose. This suggests that, within its defined scope, the app meets the expectations of its target audience. However, expanding its capabilities such as integrating teleconsultation features could extend its practical utility in digital health interventions. Overall, while the application demonstrates strong functionality, design, and content quality, improvements in engagement and subjective user experience are necessary to maximize its impact. Future iterations should focus on enhancing interactive and motivational elements to ensure long‐term usability and effectiveness.

This study possesses several notable strengths. *Peyvandi No* is an evidence‐based, interdisciplinary application developed through collaboration with experts in health sciences, psychology, and health informatics. By addressing the unique social challenges faced by cancer patients, it integrates culturally adapted educational content and interactive scenarios tailored to the Iranian context, ensuring relevance and applicability. A key strength lies in its multidimensional instructional framework, which incorporates text‐based learning, video content, real‐life experiences, interactive exercises, self‐assessment tools, and gamified components. This pedagogical design enhances engagement and facilitates experiential learning, enabling patients to develop practical social skills within a realistic yet controlled environment.

The study emphasized the interactive nature of the application, immersing users in dynamic social scenarios that allow practice and refinement of essential communication skills. By fostering self‐efficacy and reducing social isolation, *Peyvandi No*. contributes to the rehabilitation of functional social relationships—an aspect of patient care that is often underexplored but critically linked to psychological and overall well‐being. The app's adaptive feedback system provides continuous self‐evaluation and personalized guidance, supporting individualized learning trajectories and social reintegration.

Importantly, this study advances digital oncology by prioritizing functional social relationships as a fundamental component of comprehensive cancer care. Recognizing the profound impact of social connectivity on health outcomes, *Peyvandi No* is positioned as a novel and integrative intervention within digital health solutions for oncology patients.

This study also has some limitations. First, the *Peyvandi No* app does not currently support direct interaction with social relationship counselors, psychologists, or peers. Instead, patients can only submit their feedback and requests via email, which are then reviewed and referred to relevant specialists or peers by researchers. To address this limitation, future versions of the app will focus on optimizing response processes and exploring the feasibility of direct user interaction with professionals. Second, the initial version of the app was developed exclusively for the Android platform, limiting access for users of other operating systems, particularly iOS. To enhance accessibility, future plans include developing an iOS version and evaluating the possibility of a web‐based application. Third, the maximum age of participants in the usability survey was 60 years, preventing the inclusion of perspectives from older individuals. Future studies will address this gap by involving elderly users through complementary methods such as in‐depth interviews and hands‐on usability testing to gain a more comprehensive understanding of the app's accessibility for older age groups. Finally, this study did not assess the app's impact on improving patients' functional social relationships. Future research will include intervention studies to evaluate its effectiveness in enhancing the social relationships of cancer patients.

## 5 Conclusion

This study explores the development, usability evaluation, and quality assessment of *Peyvandi No*, a mobile application designed to enhance the functional social relationships of individuals with cancer. By integrating structured educational content, practical exercises, and interactive scenarios, the app aims to mitigate key social challenges encountered by patients. Preliminary evaluations indicate high levels of user satisfaction and positive expert assessments. As digital health solutions continue to evolve, the incorporation of culturally tailored technologies such as *Peyvandi No* into oncology care holds significant potential for reducing social isolation and fostering meaningful interpersonal connections among cancer patients.

## Author Contributions

**Bahare Zarei:** conceptualization, methodology, software, data curation, investigation, validation, formal analysis, visualization, resources, writing – original draft, writing – review and editing. **Masoud Bahrami:** conceptualization, methodology, investigation, validation, supervision, funding acquisition, project administration, writing – review and editing. **Hossein Beigi‐Harchegani:** conceptualization, methodology, software, data curation, validation, formal analysis, writing – review and editing. **Ashraf Kazemi:** conceptualization, methodology, data curation, validation, formal analysis, resources, writing – review and editing.

## Ethics Statement

This article was extracted from a nursing doctoral dissertation. This study received ethical approval from the Ethics Committee of IUMS, Iran (code number: IR. MUI. NUREMA. REC.1402.081). https://ethics.research.ac.ir/EthicsProposalView.php?&code=IR.MUI.NUREMA.REC.1402.081Written informed consent was obtained from the participants. The study was conducted in accordance with the Declaration of Helsinki.

## Consent

Written informed consent was obtained from the participants.

## Conflicts of Interest

The authors declare no conflicts of interest.

## Transparency Statement

The lead author Masoud Bahrami affirms that this manuscript is an honest, accurate, and transparent account of the study being reported; that no important aspects of the study have been omitted; and that any discrepancies from the study as planned (and, if relevant, registered) have been explained.

## Data Availability

All the authors have read and approved the final version of the manuscript. Masoud Bahrami had full access to all of the data in this study and took complete responsibility for the integrity of the data and the accuracy of the data analysis. All relevant data are reported in the manuscript. The data that support the findings of this study are available from the corresponding author upon reasonable request. They are not publicly available due to privacy and/or ethical restrictions. For further information, please contact the corresponding author.
